# Voice and Exercise Related Respiratory Symptoms in Extremely Preterm Born Children After Neonatal Patent Ductus Arteriosus

**DOI:** 10.3389/fped.2020.00150

**Published:** 2020-04-08

**Authors:** Merete S. Engeseth, Mette Engan, Hege Clemm, Maria Vollsæter, Roy M. Nilsen, Trond Markestad, Thomas Halvorsen, Ola D. Røksund

**Affiliations:** ^1^Department of Health and Functioning, Western Norway University of Applied Sciences, Bergen, Norway; ^2^Department of Clinical Science, University of Bergen, Bergen, Norway; ^3^Department of Pediatrics and Adolescent Medicine, Haukeland University Hospital, Bergen, Norway

**Keywords:** patent ductus arteriosus, extremely premature infant, extremely low birth weight infant, voice quality, respiratory symptoms, cohort study

## Abstract

**Objective:** To investigate voice characteristics and exercise related respiratory symptoms in extremely preterm born 11-year-old children, focusing particularly on associations with management of a patent ductus arteriosus (PDA).

**Study design:** Prospective follow-up of all children born in Norway during 1999–2000 at gestational age <28 weeks or with birthweight <1,000 g. Neonatal data were obtained prospectively on custom-made registration forms completed by neonatologists. Voice characteristics and exercise related respiratory symptoms were obtained at 11 years by parental questionnaires.

**Result:** Questionnaires were returned for 228/372 (61%) eligible children, of whom 137 had no history of PDA. PDA had been noted in 91 participants, of whom 36 had been treated conservatively, 21 with indomethacin, and 34 with surgery. Compared to the children treated with indomethacin or conservatively, the odds ratio (95% confidence interval) for the surgically treated children were 3.4 (1.3; 9.2) for having breathing problems during exercise, 16.9 (2.0; 143.0) for having a hoarse voice, 4.7 (1.3; 16.7) for a voice that breaks when shouting, 4.6 (1.1; 19.1) for a voice that disturbs singing, and 3.7 (1.1; 12.3) for problems shouting or speaking loudly. The significance of surgery *per se* was uncertain since the duration of mechanical ventilation was associated with the same outcomes.

**Conclusion:** Extremely preterm born children with a neonatal history of PDA surgery had more problems with voice and breathing during exercise in mid-childhood than those whose PDA had been handled otherwise. The study underlines the causal heterogeneity of exercise related respiratory symptoms in preterm born children.

## Introduction

Extremely preterm (EP) birth may lead to long-term complications, including respiratory problems of various etiologies (1). Survival at this early stage requires respiratory interventions, such as oxygen treatment and positive pressure ventilation, often with endotracheal intubation and mechanical ventilation (2, 3). Most of these lifesaving measures also cause various types of airway injuries with long-term consequences, challenging the diagnostic skills of health care providers (4–6).

Asthma, by far the most prevalent airway disorder, tends to be a first diagnostic option in young people with airway symptoms, often solely based on parental reports (7, 8). This practice inevitably leads to diagnostic errors, and is particularly unfortunate in EP-born individuals, given their wide causal repertoire (9–11). For example, bronchial obstruction and hyperresponsiveness are well described features after EP-birth as well as in asthma (12–16). Although linked to different immunological profiles (14, 16–19), large proportions of EP-born children are exposed to asthma medication (20). Recent literature has highlighted that also upper airway pathology creates respiratory symptoms that are misunderstood as asthma in EP-born children (21–23). We should keep in mind that the larynx is the narrowest part of the airway tree, representing a large proportion of total airway resistance (24). Thus, even minor injuries might hamper airflow when ventilatory requirements are high, such as during exercise. The larynx might be traumatized from repeated intubations or from prolonged use of mechanical ventilation (21, 25, 26). Moreover, surgical treatment of a patent ductus arteriosus (PDA) has been linked to left vocal cord paralysis (LVCP) in several studies, explained by the close proximity between the PDA and the left recurrent laryngeal nerve (11, 27, 28). Few studies have investigated symptoms of upper airway abnormalities in EP-born children (21, 26). We need more knowledge on these issues in order to develop evidence based guidelines for work-up of respiratory complaints in this group. Previous studies have suggested a role for laryngoscopy (11, 29–31); however, this notion needs support from more studies.

In this study, we used parental reports of voice abnormalities and exercise related respiratory symptoms to explore potential presence of upper airway pathology in a nationwide cohort of 11-year-old children born extremely preterm. Further, we investigated associations between these symptoms and neonatal PDA and its management.

## Materials and Methods

### Study Population

**Figure 1:**
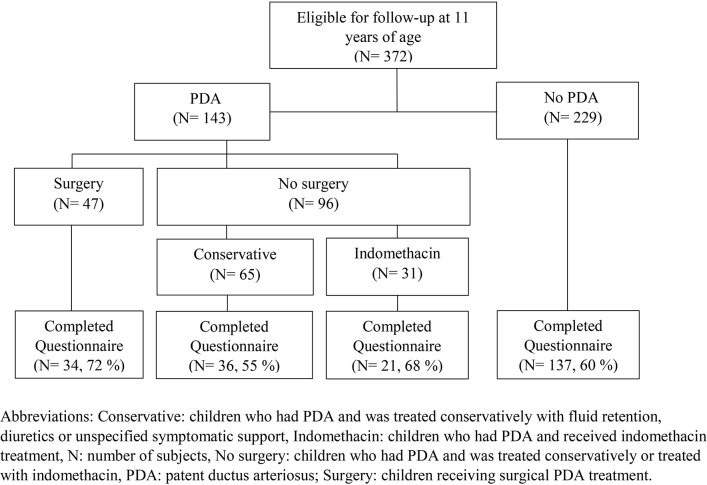
Follow-up of a national cohort of all 372 children born extremely preterm in Norway during 1999 and 2000 at a gestational age <28 weeks and/or with a birth weight <1,000 g, according to management of patent ductus arteriosus in the neonatal period.

This investigation was part of a Norwegian nationwide prospective cohort study of all children born with gestational age (GA) 23^0^–27^6^ weeks or birth weight (BW) below 1,000 g born during 1999–2000 (2). Of 638 children, 174 were stillborn or died in the delivery room, 86 died in the neonatal intensive care unit (NICU), and two declined participation (2). Three children died after discharge from NICU and one died later during the follow-up period, leaving 372 children eligible for inclusion. A “clinically significant PDA” had been diagnosed in 143 survivors, of whom 47 had undergone PDA surgery (Figure 1).

### Sources and Collection of Data

Information on the neonatal characteristic and clinical course in the NICU was obtained from compulsory notifications to the Medical Birth Registry of Norway and custom-made study registration forms (2). GA was based on ultrasound at 17–18 weeks of gestation. Small for gestational age (SGA) was defined as a BW less than the 5th^.^ percentile for GA and gender according to Norwegian growth curves (32). Bronchopulmonary dysplasia (BPD) was defined as still dependency on oxygen supplementation at 36 weeks' GA. The respiratory medical history of this cohort has been published (20, 33). PDA was diagnosed at the discretion of the participating NICUs, with algorithms based on echocardiographic assessment of the left atrium to the aortic root ratio (above 1.3–1.5 depending on clinical situation) and clinical signs as listed by Evans in 1993 (bounding hyper dynamic pulses and signs of cardiac or respiratory insufficiency) (34). Infants with a diagnosed PDA were treated either conservatively with fluid retention, diuretics, or unspecified symptomatic support, or the PDA was actively treated with either indomethacin or surgical closure performed by suture or clip.

At 11 years of age, data on previous and current symptoms were obtained from parental questionnaires. The questions: “*Does the child have breathing problems beyond what is normal during physical exertion?”* and “*Does the child make ‘scraping sounds' or other abnormal sounds from the throat during physical exertion?”* were custom made for the project. The questions regarding exercise related wheeze, asthma (ever) and use of asthma medications were obtained from the International Study of Asthma and Allergies in Childhood questionnaire (ISAAC) (35). Current asthma was defined as [1] a physician's diagnosis of asthma combined with either respiratory symptoms or use of asthma medication in the previous 12 months, *or* [2] asthma medication and symptoms in the past 12 months even if no recall of prior physician's diagnosis. Asthma medication included inhaled corticosteroids, short or long acting β2-agonists and oral leukotriene modifiers. The six questions about voice characteristics were based on the Voice Handicap Index version 5 questionnaire (36). All questions were translated to Norwegian language, and the questions analyzed in this study are described in Supplementary Table I.

### Statistical Methods

Outcome variables were voice characteristics and exercise related respiratory symptoms obtained from questionnaires at age 11 years. We investigated these binary outcomes in relation to a neonatal history of PDA vs. no PDA, and in relation to the mode of treatment in the children with a neonatal history of PDA. Those who underwent surgical closure (labeled “surgery”) were compared with those who did not undergo surgery (“no-surgery”). The “no-surgery” group included children who had been treated conservatively or with indomethacin, as initial analyses comparing the conservative and the indomethacin group showed that they did not differ in terms of neonatal variables and outcomes. Questions with graded response alternatives were transposed to dichotomous variables (no/yes) (Supplementary Table I).

Group differences were tested using independent samples *t*-test and chi-square test or Fischer‘s exact test, as appropriate. We further investigated associations by odds ratios (OR) with 95% confidence intervals (CI) using binary logistic regression. The ORs were estimated with crude and adjusted models, adjusted for days on mechanical ventilation and GA. We did not present analyses with adjustment for postnatal steroids because there is no causal link between use of postnatal steroids and PDA surgery. Potential confounders were adjusted for one by one in order to avoid that the total number of variables entered into the final regression equations exceeded 1/10 of the number of events. All analyses were performed using IBM SPSS statistics version 24 for Windows.

### Ethics

The regional ethical committee of Western Norway approved the study (REC number 2009/2271). Informed written consents were obtained from the participant's parents.

## Results

### Subjects

Questionnaires were returned for 228 of the 372 (61%) eligible children; including 91 of 143 children with a neonatal history of PDA and 137 of 228 children without PDA. Among the 91 children with PDA, 34 (37%) were treated with surgery, 36 (40%) received conservative treatment, and 21 (23%) received indomethacin (Figure 1). Six children in the surgery group had received indomethacin. Among the surgically treated children, those lost to follow-up had lower GA and BW, and spent more days on invasive mechanical ventilation. Further details on neonatal characteristics of children who were assessed and lost to follow-up at 11 years of age are presented in Supplementary Table II.

### Perinatal Characteristics

The children with a neonatal history of a PDA had lower GA, were more often intubated at birth, received more postnatal steroids and surfactant, had spent more days on continuous positive airway pressure (CPAP) and had more often developed BPD compared to children without PDA (Table 1). Those with PDA who were treated with surgical closure were born at lower GA, had spent more days on mechanical ventilation, and more often received postnatal steroids compared to the children with PDA who were not treated with surgery.

**Table 1:** Neonatal characteristics of 228 extremely premature born children (<28 weeks GA/ <1,000 g BW) participating at the follow-up at 11 years of age.

	**No PDA (*N* = 137)**	**PDA (*N* = 91)**	**PDA, no surgery (*N* = 57)**	**PDA, surgery (*N* = 34)**	**Mean difference (95% CI) No PDA vs. PDA**	***p***	**Mean difference (95% CI) no surgery vs. surgery**	***p***
**Characteristics, mean (SD)**								
GA (weeks)	27.07 (1.7)	26.07 (1.4)	26.32 (1.3)	25.6 (1.4)	1.03 (0.6–1.4)	**<0.01**	0.73 (0.15–1.31)	**0.01**
Birth weight (gram)	865 (166.1)	866 (162.8)	886 (154.5)	832 (172.9)	−0.95 (-44.6–43.0)	0.97	53.5 (-16.1–123.0)	0.13
**Start of PDA treatment, days after birth §**								
Indomethacin			10.7 (7.4)	9.5 (5.2)				
PDA Surgery				13.4 (9.6)				
**Days on invasive mechanical ventilation**								
Mean (SD)	7.1 (15.7)	11.1 (14.0)	8.1 (9.3)	16.2 (18.6)	−3.96 (−7.8–0.04)	0.05	8.1 (1.2–15.0)	**0.02**
Median (range)	2 (0–113)	6 (0–83)	5 (0–44)	10.0 (0–83)				
Days on CPAP	22.8 (18.8)	28.4 (19.4)	29.7 (19.7)	26.1 (18.8)	−5.5 (−10.5; −0.6)	**0.03**	3.69 (−4.7–12.0)	0.38
**Characteristics N (%)**								
SGA	36 (26)	8 (9)	7 (12)	1 (3)		**0.001**		0.25
Sex (female)	63 (46)	41 (45)	28 (49)	13 (38)		0.185		0.31
BPD (O_2_-suppl. at 36 weeks GA)	49 (36)	62 (68)	35 (61)	27 (79)		**<0.001**		0.08
Tracheal intubation at birth^*^	83 (64)	75 (82)	45 (79)	30 (88)		**0.001**		0.42
Surfactant	103 (75)	85 (93)	54 (95)	31 (91)		**<0.001**		0.67
Prenatal steroids	103 (75)	57 (63)	36 (63)	21 (62)		**0.04**		0.89
Postnatal steroids	37 (27)	45 (50)	23 (40)	22 (65)		**0.001**		**0.03**
Cerebral Palsy at 5 years of age	6 (4)	6 (7)	4 (7)	2 (6)		0.55		0.99

*Independent t-test, chi-square test or Fischer's exact test was used as appropriate*.

*BPD, bronchopulmonary dysplasia; CI, confidence interval; CPAP, continuous positive airway pressure. GA, gestational age; IMV, invasive mechanical ventilation; PDA, patent ductus arteriosus; SD, standard deviation; SGA, small for gestational age*.

* *Missing data: Tracheal intubation at birth: Eight cases missing from the “no PDA” group and two cases missing from the “PDA”/“PDA, no surgery” groups. §Start of PDA treatment: Data are missing for nine children regarding age at day of PDA surgery. 21 children in the “PDA, no surgery” group received indomethacin treatment and six children in the “PDA, surgery” group received indomethacin treatment before surgery. Values in bold indicates a p-value of less than 0.05*.

### Questionnaire Based Data of Respiratory and Voice Related Symptoms at 11 Years of Age

**Figure 2:**
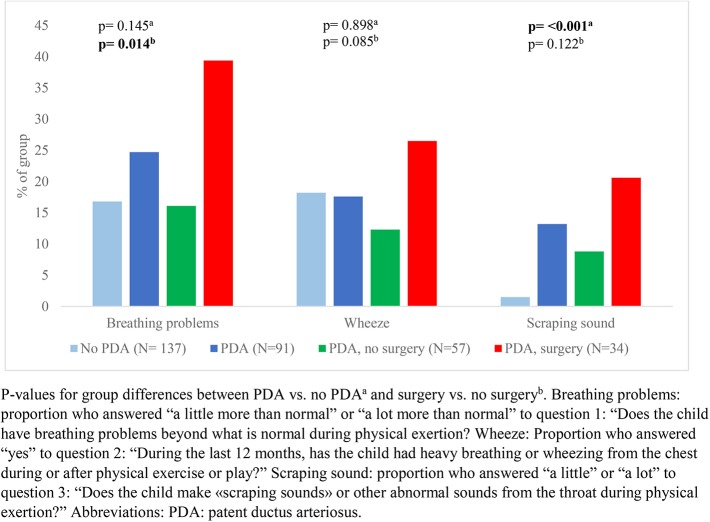
Reported respiratory symptoms during or after physical activity among a national cohort of extremely preterm born children at 11 years of age according to diagnosis and treatment of patent ductus arteriosus in the neonatal period.

**Figure 3:**
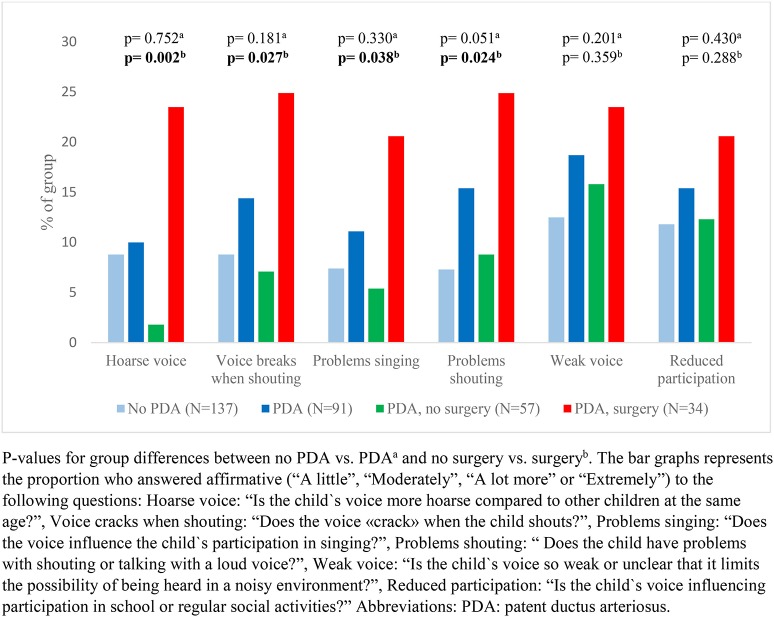
Reported voice characteristics among a national cohort of extremely preterm born children at the age of 11 years according to diagnosis and management of patent ductus arteriosus in the neonatal period.

**Figure 4:**
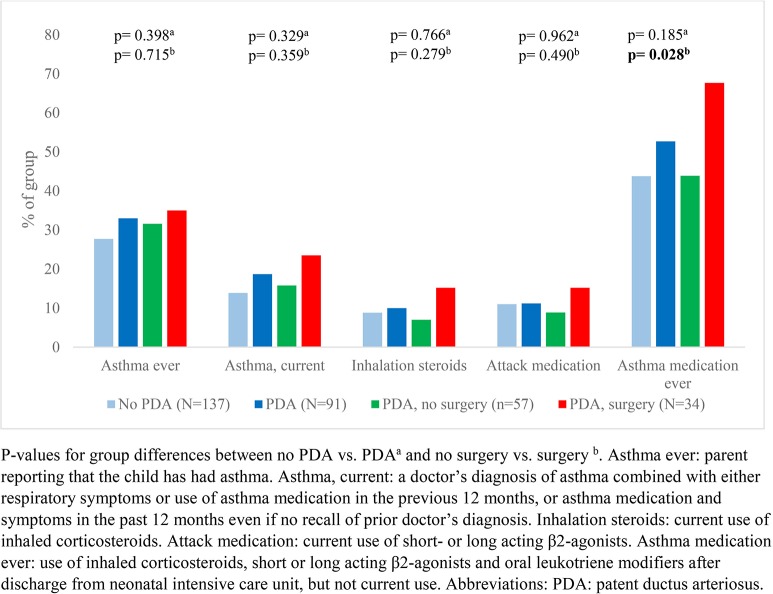
Reports of asthma and use of asthma medications among a national cohort of extremely preterm born children at 11 years of age according to diagnosis and treatment of patent ductus arteriosus in the neonatal period.

Our data showed that exercise related respiratory symptoms or voice problems were more common in children with- than without a neonatal history of PDA. However, differences were only statistically significant for scraping sound during physical exertion (Figures 2, 3). There were no important differences in reports of asthma or use of asthma medications (Figure 4).

In children who had a neonatal history of PDA, surgical closure was associated with more frequent reports of breathing problems during physical exertion (Figure 2) and voice related symptoms (Figure 3) compared to the no-surgery group. There were no differences between the surgery group and the no-surgery group regarding current asthma, current use of asthma medication or ever having had asthma, but a higher proportion of the surgery group had previously received asthma medication compared to the no-surgery group (Figure 4). In total, one or more symptoms related to respiration during exertion or voice were reported for 20 (61%) of the participants in the surgery group and 17 (31%) in the no-surgery group (*p* = 0.006).

### Logistic Regression Analyses

#### Exercise Related Respiratory Symptoms

The odds ratio (OR) for having scraping sounds during physical exertion was increased among children with a PDA diagnosis (OR: 10.25; 95% CI: 2.24–47.0; *p* = 0.03) compared to the group without PDA, but not for other symptoms (not shown in tables). Among children with PDA, the crude odds of having breathing problems during physical exertion were higher in the surgery group relative to the no-surgery group (Table 2). After adjustment for number of days on mechanical ventilation, the OR of having breathing problems during physical exertion was still increased, but confidence intervals were wide. Adjusting for GA did not have impact on the OR.

**Table 2:** The odds ratio of having respiratory symptoms during or after physical activity at 11 years of age in a national cohort of extremely preterm born children according to treatment for patent ductus arteriosus (no surgery vs. surgery) in the neonatal period.

	**PDA treatment**	***N* (%)**	**Crude OR (95% CI)**	***p***	**aOR1 (95% CI)**	***p***	**aOR2 (95% CI)**	***p***
Breathing problems	No surgery	9/56 (16)	3.4 (1.3–9.2)	**0.02**	2.6 (0.9–7.4)	0.08	3.2 (1.1–9.0)	**0.03**
	Surgery	13/33 (39)						
Wheeze	No surgery	7/57 (12)	2.6 (0.9–7.7)	0.09	2.5 (0.8–7.9)	0.11	2.8 (0.9–8.8)	0.08
	Surgery	9/34 (27)						
Scraping sound	No surgery	5/57 (9)	2.7 (0.8–9.3)	0.12	1.8 (0.5–6.8)	0.41	2.4 (0.7–8.7)	0.17
	Surgery	7/34 (21)						

*Breathing problems: proportion who answered “a little more than normal” or “a lot more than normal” to question 1: “Does the child have breathing problems beyond what is normal during physical exertion?” Wheeze: Proportion who answered “yes” to question 2: “During the last 12 months, has the child had heavy breathing or wheezing from the chest during or after physical exercise or play?” Scraping sound: proportion who answered “a little” or “a lot” to question 3: “Does the child make ≪scraping sounds≫ or other abnormal sounds from the throat during physical exertion?” aOR1, adjusted for total number of days on invasive mechanical ventilation. aOR2, adjusted for gestational age; CI, confidence interval; OR, odds ratio; PDA, patent ductus arteriosus. Values in bold indicates a p-value of less than 0.05*.

#### Voice Characteristics

The OR for having symptoms related to voice was not increased among children with a PDA diagnosis, compared to the group without PDA. Among children with PDA, the crude odds of having a hoarse voice, a voice that breaks when shouting, a voice that affects participation in singing or a voice that leads to problems talking loudly/shout, were higher in the surgery group compared to the no-surgery group (Table 3). Adjusted for number of days on mechanical ventilation, the ORs were no longer increased for any of the voice characteristics. Adjusted for GA, the ORs were still increased for having a hoarse voice or a voice that cracks when shouting.

**Table 3:** The odds ratio of having voice symptoms at 11 years of age in a national cohort of extremely preterm born children according to treatment (no surgery vs. surgery) for patent ductus arteriosus in the neonatal period.

	**PDA treatment**	***N* (%)**	**Crude OR (95% CI)**	***p***	**aOR1 (95% CI)**	***p***	**aOR2 (95% CI)**	***p***
Hoarse voice	No surgery	1/56 (2)	16.9 (2.0–143)	**0.009**	9.6 (1.0–93.8)	0.05	14.1 (1.6–122)	**0.02**
	Surgery	8/34 (24)						
Voice cracks when the child shouts	No surgery	4/56 (7)	4.7 (1.3–16.7)	**0.017**	3.2 (1.2–20.7)	0.10	3.9 (1.1–14.4)	**0.04**
	Surgery	9/34 (27)						
Voice influences participation in singing	No surgery	3/56 (5)	4.6 (1.1–19.1)	**0.04**	2.8 (0.6–13.1)	0.19	3.7 (0.9–16.2)	0.08
	Surgery	7/34 (21)						
Problems shouting or talking loudly	No surgery	5/57 (9)	3.7 (1.1–12.3)	**0.03**	2.6 (0.7–9.3)	0.14	3.2 (0.9–10.9)	0.07
	Surgery	9/34 (27)						
Weak or unclear voice	No surgery	9/57 (16)	1.6 (0.6–4.8)	0.36	1.0 (0.3–3.4)	0.96	1.5 (0.5–4.6)	0.46
	Surgery	8/34 (24)						
Voice influences participation in school or social activities	No surgery	7/57 (12)	1.9 (0.6–5.8)	0.29	1.3 (0.4–4.4)	0.73	1.8 (0.5–5.8)	0.35
	Surgery	7/34 (21)						

*N (%) represents proportion who answered affirmative (“A little,” “Moderately,” “A lot more” or “Extremely”) to the following questions: Hoarse voice: “Is the child‘s voice more hoarse compared to other children at the same age? Voice cracks when the child shouts: Does the voice ≪crack≫ when the child shouts?,” Voice influences participation in singing: “Does the voice influence the child‘s participation in singing?,” Problems shouting or talking loudly: “Does the child have problems with shouting or talking with a loud voice?,” Weak or unclear voice: “Is the child‘s voice so weak or unclear that it limits the possibility of being heard in a noisy environment?,” Voice influences participation in school or social activities: “Is the child‘s voice influencing participation in school or regular social activities?.” aOR1, adjusted for total number of days on invasive mechanical ventilation; aOR2, adjusted for gestational age; CI, confidence interval; OR, odds ratio; PDA, patent ductus arteriosus. Values in bold indicates a p-value of less than 0.05*.

## Discussion

We found that voice and exercise related respiratory symptoms were more common in 11-year-old children born extremely preterm with a neonatal history of PDA surgery, compared to children whose PDA had been managed otherwise. However, the significance of surgery *per se* remains uncertain since adjusting for days of mechanical ventilation significantly weakened the associations.

Previous studies have described that EP-born children with left vocal fold paralysis (LVCP) following PDA surgery had prolonged dependency of mechanical ventilation compared to surgically treated children without LVCP (11, 28, 37, 38). Unfortunately, the respective duration of mechanical ventilation before vs. after surgery could not be estimated in our study. Thus, prolonged mechanical ventilation in the surgery group could be *caused* by a possible LVCP or other complications following surgery (39). However, it could also reflect *confounding by indication*; i.e., those treated surgically were also those with the most severe disease, therefore spending most days ventilated (40). Both mechanisms might have been operative, but we do not have data to disentangle this scenario. Days on mechanical ventilation was associated with symptoms in all subgroups, also in those with no history of PDA, although at a lower odds ratio. The observed association (co-linearity) between days on mechanical ventilation and surgical PDA treatment complicates interpretations of the regression models. We therefore report both adjusted and unadjusted data in Tables 2, 3. However, taken together the data indicate that neonatal PDA surgery leads to more voice and exercise related respiratory symptoms in mid-childhood, possibly influenced also by prolonged mechanical ventilation.

Management of PDA in EP-born neonates is debated. Options include a conservative approach, pharmacologic intervention, or surgical ligation, the latter usually used as a last resort (41, 42). Knowledge on long-term outcomes must count in these discussions. There are few studies reporting on voice characteristics and exercise related respiratory symptoms in children and adults exposed to neonatal PDA surgery (30). Although LVCP is a well-described complication, an unknown fraction may pass unnoticed or are misinterpreted during the neonatal period as symptoms may be vague, transient and uncharacteristic (27, 29, 37, 38, 43). Importantly, we know that pediatric LVCP does not usually recover (11, 44–46). Therefore, symptoms may continue to be overlooked or erroneously related to other disorders or even to malingering, later in life. We do not have research based data to substantiate this notion. However, early life events are rarely considered by respiratory specialists (5), and LVCP is probably not on top of the physicians‘ list when trying to interpret airway symptoms. In a regional study of 11 EP born adults who had undergone PDA surgery in the 1980s, seven had LVCP of whom six reported trouble with their voice. None of them were comfortable with singing or speaking loudly, and all disclosed prolonged inspirium, wheeze or stridor when tested on a treadmill (11). Importantly, three of them had a long history of “difficult-to-treat asthma,” but they could substantially reduce medication after receiving the LVCP diagnosis. In our study, we found no association between PDA surgery and current asthma medication; however, a higher proportion of the surgically treated children had used asthma medication. Thus, breathing problems could initially have been perceived as asthma, and medication subsequently stopped due to lack of effect.

In the present study, we also identified children with respiratory and voice related symptoms among children with no history of PDA, or who had not undergone PDA surgery, implying that these symptoms are of multi-factorial origin. Prolonged mechanical ventilation is associated with subglottic stenosis and injury to the vocal cords (47), which may lead to stridor and affect voice (48). Walz et al. (25) found that prolonged intubation (more than 4 weeks) was associated with long-term reduced voice quality. In univariate analysis, both the presence of a PDA and surgical closure of a PDA were associated with lower score on voice related quality of life. In multivariate analysis, PDA did not contribute to the model, but PDA surgery was not included. French et al. (26) reported that 58% of school aged children born before 25 weeks' gestation had moderate to severe hoarseness, and that the number of intubations (more than five) was associated with voice disorders. The authors suggested that the voice abnormalities could be related to laryngeal injury from endotracheal intubation. As only three of the 67 tested children had undergone PDA surgery, they argued that surgery could not have contributed to the voice problems in their cohort (26). Simpson et al. (21) found that 25/35 very preterm born children presented with dysphonia at 11 years of age, and increased dysphonia severity was predicted by lower GA, increased number of intubations and days of mechanical ventilation. Only three subjects had undergone PDA surgery, but they represented 3/14 subjects with moderate to severe dysphonia. Presence of dysphonia was associated with reports of previous wheeze, asthma diagnosis and former use of asthma medications. However, there was no difference in lung function between groups with- or without dysphonia, and the authors suggested upper airway pathology and dysfunctional breathing contributing to increased reports of respiratory symptoms (21).

The major limitations of this study were the relatively small sample size and the fact that we relied on parental observation of respiratory and voice related symptoms. We know from asthma research that there are differences between parents' and children's perceptions (49). However, in this age group parental information is usually what we have to go by in clinical work. As in all follow-up studies, attrition influences interpretation; in our case underlined by differences between responders and non-responders listed in the appendix. Importantly, laryngoscopies had not been performed, precluding knowledge as to whether LVCP, or other laryngeal abnormalities contributed to the increased odds of respiratory and voice related symptoms in the PDA surgery group. Inability to ascertain why infants exposed to PDA surgery needed more days on mechanical ventilation challenged attempts to interpret the role of this variable in the regression models and thus in the causal chain leading to symptoms.

## Conclusion

Extremely preterm born school-children who had undergone neonatal PDA surgery had more voice and exercise related respiratory symptoms than children exposed to other modes of PDA treatment. Although these symptoms are likely to have a compound etiology, when present in children exposed to neonatal PDA surgery, they must prompt a search for upper airway abnormalities, and not lead to empirical prescription of asthma medication. We must not forget early life events when dealing with respiratory symptoms.

## Data Availability Statement

Data from the study are available upon request. There are legal restrictions on sharing these data publicly due to the data containing sensitive and identifiable information. The data set contains information like birthweight, gestational age, birth data and gender - information that may be used to directly identify individuals as Norway is a small country and as extremely preterm birth applies to relatively few individuals in each hospital each year. In the informed consents signed by the guardians of the participants of this study, and granted by the regional committee for medical ethics in Helse Vest, guardians were not asked about data sharing.

## Ethics Statement

The studies involving human participants were reviewed and approved by The Regional Commitee for Medical and Health Research Ethics west (REC number 2009/2271). Written informed consent to participate in this study was provided by the participants' legal guardian/next of kin.

## Author's Note

Preliminary data from this study was presented as an abstract at The European Respiratory Congress in 2018, where copyright is retained by the author or employer [as part of the conditions of the author(s)'s employment].

## Author Contributions

TH, TM, OR, MV, and HC designed the data collection instruments, collected the data, contributed to interpretation of the data, and reviewed and revised the manuscript for important intellectual content. ME contributed to the analyses and interpretation of the data, and reviewed and revised the manuscript for important intellectual content. RN contributed to the statistical analyses and interpretation of the data, and reviewed and revised the manuscript for important intellectual content. MSE carried out the statistical analyses and the interpretation of the data, drafted the article, and reviewed and revised the manuscript. All authors approved the final manuscript as submitted and agree to be accountable for all aspects of the work.

### Conflict of Interest

The authors declare that the research was conducted in the absence of any commercial or financial relationships that could be construed as a potential conflict of interest.
