# Supplementary material for: Voice and Exercise Related Respiratory Symptoms in Extremely Preterm Born Children After Neonatal Patent Ductus Arteriosus
Source: Front Pediatr. 2020 Apr 8;8:150. doi: 10.3389/fped.2020.00150 (PMC7156623; doi:10.3389/fped.2020.00150)
Supplement: Supplementary file 1 [file Table_1.DOCX]

| **Respiration during/after physical activity** | Answers | No surgery (N=57) | Surgery  (N=34) | Missing N (%) |
| --- | --- | --- | --- | --- |
| 1. Does the child have breathing problems beyond what is normal during physical exertion? | ^0^No  ^1^A little more than normal  ^1^A lot more than normal | 47  9 | 20  13 | 54 (37.8) |
| 1. During the last 12 months, has the child had heavy breathing or wheezing from the chest during or after physical exercise or play? | ^0^No  ^1^Yes | 50  7 | 25  9 | 52 (36.4) |
| 1. Does the child make «grinding sounds» or other abnormal sounds from the throat during physical exertion? | ^0^No  ^1^A little  ^1^A lot | 52  5  0 | 27  6  1 | 52 (36.4) |
| **Voice** | | | | |
| 1. Is the child`s voice more hoarse compared to other children at the same age? | ^0^Not at all  ^1^A little  ^1^Moderately  ^1^A lot more  ^1^Extremely | 55  0  1  0  0 | 26  6  0  2  0 | 53 (37.1) |
| 1. Does the voice «break» when the child shouts? | ^0^Not at all  ^1^A little  ^1^Moderately  ^1^A lot more  ^1^Extremely | 52  3  1  0  0 | 25  8  0  1  0 | 53 (37.1) |
| 1. Does the voice influence the child`s participation in singing? | ^0^Not at all  ^1^A little  ^1^Moderately  ^1^A lot more  ^1^Extremely | 53  2  1  0  0 | 27  4  3  0  0 | 53 (37.1) |
| 1. Does the child have problems with shouting or talking with a loud voice? | ^0^Not at all  ^1^A little  ^1^Moderately  ^1^A lot more  ^1^Extremely | 52  4  1  0  0 | 25  5  2  2  0 | 52 (36.4) |
| 1. Is the child`s voice so weak or unclear that it limits the ability of being heard in a noisy environment? | ^0^Not at all  ^1^A little  ^1^Moderately  ^1^A lot more  ^1^Extremely | 48  7  1  1  0 | 26  5  0  3  0 | 52 (36.4) |
| 1. Is the child`s voice influencing participation in school or regular social activities? | ^0^Not at all  ^1^A little  ^1^Moderately  ^1^A lot more  ^1^Extremely | 50  6  0  1  0 | 27  4  1  1  1 | 52 (36.4) |

Supplementary Table I Questions about respiration during or after physical activity and voice

symptoms.

Abbreviations: Missing: Representing how many of the 143 eligible children with PDA who did not respond to the question, 0: Coded as “No” or absence of characteristic in statistical analysis, 1: Coded as “Yes” (all degrees of yes) or presence of characteristic in statistical analysis. The questions were handed out in Norwegian language in this study, but they were translated back to English for the readers of this publication.
