# Supplementary material for: Voice and Exercise Related Respiratory Symptoms in Extremely Preterm Born Children After Neonatal Patent Ductus Arteriosus
Source: Front Pediatr. 2020 Apr 8;8:150. doi: 10.3389/fped.2020.00150 (PMC7156623; doi:10.3389/fped.2020.00150)
Supplement: Supplementary file 2 [file Table_2.docx]

|  | | No PDA (N=229) | | | | PDA (N=143) | | | | PDA, no surgery (N=96) | | | | PDA surgery (N=47) | | | |
| --- | --- | --- | --- | --- | --- | --- | --- | --- | --- | --- | --- | --- | --- | --- | --- | --- | --- |
|  | Participated at 11 years (N=137) | | Lost to follow-up (N=92) | MD  (95%CI) | p | Participated at 11 years (N=91) | Lost to follow-up (N=52) | MD  (95%CI) | p | Participated at 11 years (N=57) | Lost to follow-up (N=39) | MD  (95%CI) | p | Participated at 11 years (N=34) | Lost to follow-up (N=13) | MD  (95%CI) | p |
| Characteristics, mean (SD) | | | | | | | | | | | | | | | | | |
| GA (weeks) | | 27.1  (1.7) | 26.6  (1.8) | 0.4  (-0.0 - 0.9) | 0.06 | 26.0  (1.4) | 25.7  (1.3) | 0.33  (-0.14 - 0.80) | 0.17 | 26.3  (1.3) | 26.1  (1.2) | 0.21  (-0.32 - 0.74) | 0.43 | 25.6  (1.35) | 24.5  (1.05) | 1.1  (0.2 - 1.9) | **0.02** |
| BW (gram) | | 865  (166) | 857  (179) | 8  (-38 - 53) | 0.72 | 866  (163) | 834  (184) | 32  (-27 - 90) | 0.29 | 886  (155) | 888  (170) | -2  (-69 - 64) | 0.95 | 832  (173) | 673  (124) | 159  (53 - 265) | **0.004** |
| Days on IMV | | 7.1  (15.7) | 7.3  (16.0) | 0.2  (-4.4 - 4.0) | 0.94 | 11.1  (14.0) | 14.5  (19.8) | -3.37  (-8.99 - 2.24) | 0.24 | 8.1  (9.3) | 7.7  (10.6) | 0.38  (-3.67 - 4.4) | 0.85 | 16.2  (18.6) | 34.8  (26.9) | 18.7  (4.8 - 32.5) | **0.01** |
| Days on CPAP | | 22.8  (18.2) | 24.2  (19.6) | 1.4  (-6.3 - 3.6) | 0.60 | 28.4  (19.4) | 26.4  (18.4) | 1.92  (-4.62 - 8.46) | 0.56 | 29.7  (19.7) | 22.7  (15.0) | 7.02  (-0.02 - 14.06) | 0.05 | 26.1  (18.8) | 37.6  (23.6) | 11.6  (-24.8 - 1.7) | 0.09 |
| Characteristics, N (%) | | | | | | | | | | | | | | | | | |
| SGA | | 36 (26) | 20 (22) |  | 0.43 | 8 (9) | 5 (10) |  | 0.99 | 7 (12) | 3 (8) |  | 0.74 | 1(3) | 2 (15) |  | 0.18 |
| Sex (female) | | 74 (54) | 37 (40) |  | **0.04** | 41 (45) | 20 (39) |  | 0.44 | 28 (49) | 16 (41) |  | 0.43 | 13 (38) | 4 (31) |  | 0.74 |
| BPD | | 49 (36) | 33 (36) |  | 0.99 | 62 (68) | 21 (40) |  | **0.001** | 35 (61) | 9 (23) |  | **<0.001** | 27 (79) | 12 (92) |  | 0.41 |
| Tracheal intubation (birth)* | | 83 (64) | 45 (52) |  | 0.06 | 75 (84) | 44 (86) |  | 0.75 | 45 (82) | 31 (82) |  | 0.98 | 30 (88) | 13 (100) |  | 0.56 |
| Surfactant | | 103 (75) | 60 (65) |  | 0.10 | 85 (93) | 48 (92) |  | 0.99 | 54 (95) | 35 (90) |  | 0.44 | 31 (91) | 13 (100) |  | 0.55 |
| Prenatal steroids | | 103 (75) | 63 (69) |  | 0.27 | 57 (63) | 34 (65) |  | 0.74 | 36 (63) | 23 (59) |  | 0.68 | 21 (62) | 11 (85) |  | 0.18 |
| Postnatal steroids | | 37 (27) | 23 (25) |  | 0.74 | 45 (50) | 24 (46) |  | 0.70 | 23 (40) | 12 (31) |  | 0.34 | 22 (65) | 12 (92) |  | 0.08 |
| Cerebral Palsy  GMFCS level 1  2  3  4  5 | | 6 (4)  4  0  2  0  0 | 9 (10)  3  1  1  1  3 |  | 0.11 | 6 (7)  0  2  1  2  1 | 8 (15)  2  2  1  1  2 |  | 0.09 | 4 (7)  0  2  0  2  0 | 4 (10)  0  2  1  0  1 |  | 0.71 | 2 (6)  0  0  1  0  1 | 4 (31)  0  2  0  2  0 |  | **0.04** |

Supplementary Table II Neonatal characteristics of the 372 extremely premature born children (<28 weeks GA/<1000g BW) participating in the study or lost to follow-up at 11 years of age.

Independent t-test, chi-square test or Fischer`s exact test was used as appropriate. Abbreviations: BPD: bronchopulmonary dysplasia (i.e. oxygen supplement at gestational age 36 weeks), BW: birth weight, CI: confidence interval, CPAP: continuous positive airway pressure. GA: gestational age, GMFCS: Gross Motor Function Classification System (1-5), IMV: invasive mechanical ventilation, MD: mean difference, PDA: patent ductus arteriosus, SD: standard deviation, SGA: small for gestational age, *Missing data: Tracheal intubation at birth: Eight cases from “no PDA, participated”, five cases from “no PDA, lost to follow-up”, two cases from “no surgery, participated”, and one case from “no surgery, lost to follow-up”.
